# Transcriptional Regulation of *INSR*, the Insulin Receptor Gene

**DOI:** 10.3390/genes10120984

**Published:** 2019-11-29

**Authors:** Sandhya Payankaulam, Ana-Maria Raicu, David N. Arnosti

**Affiliations:** 1 Department of Biochemistry and Molecular Biology, Michigan State University, 603 Wilson Rd. 413 Biochemistry, East Lansing, MI 48824, USA; sandhya@msu.edu; 2 Cell and Molecular Biology Program, Michigan State University, 603 Wilson Rd. 413 Biochemistry, East Lansing, MI 48824, USA; raicuana@msu.edu

**Keywords:** insulin receptor, IR, *INSR*, transcriptional regulation

## Abstract

The insulin receptor gene encodes an evolutionarily conserved signaling protein with a wide spectrum of functions in metazoan development. The insulin signaling pathway plays key roles in processes such as metabolic regulation, growth control, and neuronal function. Misregulation of the pathway features in diabetes, cancer, and neurodegenerative diseases, making it an important target for clinical interventions. While much attention has been focused on differential pathway activation through ligand availability, sensitization of overall signaling may also be mediated by differential expression of the insulin receptor itself. Although first characterized as a “housekeeping” gene with stable expression, comparative studies have shown that expression levels of the human *INSR* mRNA differ by tissue and in response to environmental signals. Our recent analysis of the transcriptional controls affecting expression of the Drosophila insulin receptor gene indicates that a remarkable amount of DNA is dedicated to encoding sophisticated feedback and feed forward signals. The human *INSR* gene is likely to contain a similar level of transcriptional complexity; here, we summarize over three decades of molecular biology and genetic research that points to a still incompletely understood regulatory control system. Further elucidation of transcriptional controls of *INSR* will provide the basis for understanding human genetic variation that underlies population-level physiological differences and disease.

## 1. Insulin Receptor and Insulin Signaling

The insulin signaling pathway in metazoans regulates diverse processes including cell growth and metabolic homeostasis. These critical processes are mediated by the insulin receptor (IR), a single pass transmembrane receptor with tyrosine kinase activity. Upon binding of insulin, a host of further signals are transmitted inside the cell, leading to changes in glucose transport, gene expression, and proliferation. Since the discovery of this membrane receptor, we have gained extensive insight into the biological function of its role in development, physiology, and disease. At the same time, the detailed molecular underpinnings of the signaling pathway have been elucidated, in part through studies of model organisms including *Drosophila melanogaster*, *Caenorhabditis elegans,* and the mouse. The human *INSR* gene, located on chromosome 19, encodes the insulin receptor, a heterotetrameric glycoprotein found in the membrane of most cells of the human body. IR encodes an alpha and beta subunit, which are proteolytically cleaved after dimerization with another alpha-beta pair. The two dimers are linked via disulfide bonds to create a heterotetrameric protein of approximately 450 kDa in mass, not considering post-translational glycosylation. The two extracellular alpha subunits bind insulin, while the two beta subunits traverse the cell membrane and harbor intracellular tyrosine kinase domains [1,2,3].

In response to high glucose levels in the blood after a meal, insulin is released by pancreatic beta cells to signal tissues to take up blood glucose and metabolize it. Insulin binding to the insulin receptor induces a conformational change in the alpha subunits of the receptor, leading to conformational changes in the intracellular beta subunits. The active sites of the beta subunits come into close contact with each other and trans-phosphorylate neighboring tyrosine residues. These phosphorylated tyrosine residues serve as binding sites for adaptor proteins involved in transducing the signal through the cell. Auto-phosphorylation first allows for the binding of adaptor proteins, including insulin receptor substrate 1 (IRS1) to the intracellular phosphorylated tyrosine residues, leading to phosphorylation of this signal mediator. IRS proteins are involved in activating two downstream signaling pathways: the phosphatidylinositol 3-kinase (PI3K)/AKT pathway, which is important for insulin’s metabolic activity, and the Ras-mitogen-activated protein kinase (MAPK) pathway, which is responsible for cell growth and development [2,3].

Activation of the PI3K/AKT pathway is initiated by PI3K binding to phosphorylated IRS-1. The active site of the activated PI3K moves in close proximity to the lipid membrane and phosphorylates phosphoinositides found in the cell membrane, such as PIP2, to produce PIP3, which binds to the PIP3-dependent protein kinase (PDK1). In turn, PDK1 activation results in the activation of Akt (also known as protein kinase B), a diffusible cytoplasmic kinase. Akt is a key signaling molecule that mediates the effect of insulin, stimulating the movement of glucose membrane transporters to the cell membrane, which increases glucose uptake from the blood into the cell. Akt also phosphorylates enzymes necessary for converting glucose to glycogen. Other downstream effectors of Akt include the target of rapamycin kinase (mTOR) and the forkhead-related FOXO transcription factors FOXO1, FOXO3a, and FOXO4 [4]. FOXO transcription factors are phosphorylated by Akt on three conserved serine and threonine residues, which leads to their retention in the cytoplasm and downregulation of FOXO transcriptional targets [2,4,5]. Alternatively, IR activation can lead to a signaling cascade involving the MAPK signaling pathway, which ultimately also leads to the activation of mTOR and other transcription factors [6]. These insulin receptor-mediated signaling pathways are highly conserved across metazoans, with homologs to IR, IRS-1, PI3K, FOXO, and other proteins found in *C. elegans*, mouse, and Drosophila [7].

In mammals, changes in the overall levels of insulin receptor have been shown to be of physiological importance. At its most extreme, IR levels are severely impacted in certain disease models, such as in the *ob/ob* leptin mutant mouse model [8]. Heterozygous *INSR* mutant mice are largely normal in terms of growth and fertility but have defects in downstream signaling [9]. In humans, heterozygous carriers of *INSR* null mutations exhibit abnormal glucose tolerance, indicating that gene dosage and expression are important [10]. However, the significance of transcriptional regulation for this gene is only now starting to come into view, and this review will summarize convergent lines of evidence that lend urgency to deciphering this important but little-explored level of regulation for the key receptor of an ancient metazoan pathway.

## 2. The Human Insulin Receptor Gene

The human insulin receptor gene spans ~180 kb and is composed of 22 exons; the first 11 exons encode the extracellular alpha subunit and the remaining 11 exons encode the intracellular beta subunit. Cloning of *INSR* cDNA was first reported in 1985 by two groups [11,12]. The predicted transcript lengths differed by 36 bp, which reflects alternative splicing of exons 11. This splicing event impacts the C terminus of the alpha subunit [13]; the isoform excluding this exon encodes a 1370 residue protein termed IR-A, while the 1382 residue isoform is termed IR-B. These isoforms are differentially expressed in tissues, throughout developmental stages, and in disease states [2,14]. They also play unique roles in the body: IR-A promotes cell growth, in particular during fetal development, and is also found in the brain and spleen. IR-B is expressed more highly in differentiated cells such as pancreatic beta cells, liver, muscle, adipose tissue, and kidney cells [15,16,17]. Additionally, IR-B is the predominant regulator of glucose homeostasis and has two-fold higher tyrosine kinase activity, making it more involved in metabolic signaling than isoform A. The Ras-MEK1-ERK pathway can regulate this alternative splicing event and may impact inclusion of exon 11 to influence IR-B isoform levels [14].

**Figure 1:**
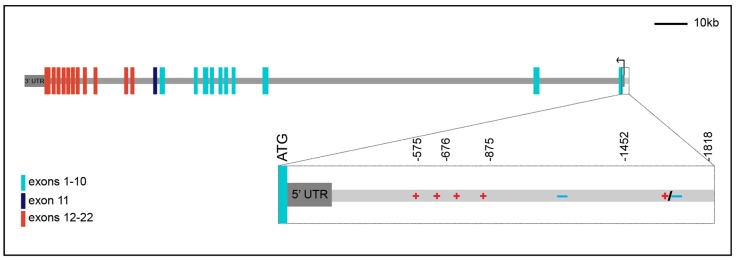
Schematic representation of the human *INSR* gene spanning ~180 kb. The single black arrow represents two transcriptional start sites at -40 and -144 from ATG with the 144 bp 5′ UTR indicated in dark gray. There are two alternative 3′ UTRs of ~450 bp and ~4.5 kb based on GENCODE data but only a single ~4.5 kb UTR (shown) based on RefSeq annotation. Colored bars indicate exons: light blue bars correspond to exons 1–10, which encode the alpha subunit of the protein. Red bars indicate exons 12–22, which encode the beta subunit of the protein. The single dark blue bar indicates the alternatively spliced exon 11, which is retained in *INSR-B* but is missing from *INSR-A*. All coordinates are relative to ATG. Magnified view of the 2 kb region directly upstream of the ATG combines data from 18,19,21, and 23–26, indicating positively acting regulatory elements present from −575 to −875, negative regulatory elements directly 5′ of that, and tissue-specific positive or negative regulatory elements 5′ of −1452.

In addition to alternative splicing of the *INSR* transcript, the isoforms differ in the length of their 5′ and 3′ untranslated regions (UTRs). Original studies mapping the transcriptional start site (TSS) of *INSR* failed to come to a consensus about the exact location of transcriptional initiation, with several TSSs mapped within a few hundred base pairs of the ATG codon [13,18,19,20,21]. Recent GENCODE and RefSeq annotations have mapped only two transcriptional start sites, one 144 bp and the other 40 bp 5′ of the ATG. In addition, there are alternative 3′UTRs of ~450 bp and ~4.5 kb (Figure 1). The biological importance of the alternative start and polyadenylation sites and their potential impact on isoform-specific regulation and expression has not been further explored.

When it was first cloned, the *INSR* gene was termed a housekeeping gene based on its widespread pattern of expression. The structure of the basal promoter region supported that conclusion, as it conformed to early concepts of housekeeping gene characteristics, including an Sp1-binding, GC-rich promoter proximal sequence, and lack of a TATA box [21]. However, even in the early studies, it was found that steady-state levels of the *INSR* transcript varied in response to physiological signals. More recently, the identification of complex cis-regulatory wiring associated with this gene have challenged the idea that *INSR* is a housekeeping gene without much dynamic regulation [22]. Evidence for dynamic transcriptional regulation of *INSR* is supported by studies investigating cis-regulatory elements found both within the promoter and intronic regions of the gene, which exhibit cell-type specific activities [22]. Differences in response to insulin, dexamethasone, and other treatments in various cell lines also indicate complex cell-type regulation of the *INSR* gene [23]. Finally, the differential expression of IR isoforms in some diseases further underlines the importance of understanding dynamic cis-regulation of this gene, as discussed below.

## 3. Promoter Analysis

Transcriptional analysis of the *INSR* gene commenced at a time when there was little appreciation for the role of distal enhancers, and most studies focused on a 2 kb region 5′ of the *INSR* translational start site. Initial studies using primer extension assays and S1 nuclease mapping determined the existence of several TSSs with inconsistent results. RefSeq gene predictions from NCBI now suggest a single TSS at -144 while GENCODE indicates a second TSS -40 upstream of ATG. Initial experiments tested portions of the 2 kbp region 5′ of the TSS using chloramphenicol acetyltransferase reporters in diverse cell lines. Results from a combination of studies spanning almost two decades of work indicate that this promoter fragment has activity in the cell lines tested, with some evidence for tissue-specific elements, containing both activating and repressing functions (Figure 1).

These studies tested the human *INSR* promoter in both human and non-human mammalian cell lines. To identify functional elements in this promoter fragment, different segments were assayed in the HepG2 human hepatoblastoma cell line [19,21,24,25,26,27], the MCF-7 human breast cancer cell line [21,26], the 3T3 human fibroblast cell line [21], the cervical carcinoma HeLa cell line [27], and the HEK293 human embryonic kidney cell line [21]. Segments were also tested in CHO (Chinese hamster ovary) cells [18,25,28], COS (monkey fibroblast-like) cells [28], and CV1 monkey kidney cells [19]. These experiments indicate that in most cell types, regions of the promoter from -500 to -875 interact with transcriptional activators to provide most of the activity. Additional positive and negative regulatory elements also appear to be present in more 3′ and 5′ regions. Even in this discrete element, tissue-specific differences are found in the ~1 kb region 5′ of -875; this portion of the promoter appears to contain activation potential in HepG2 cells and repression activity in MCF-7 cells.

These early promoter studies provided little information about the transcription factors involved in this widespread activity or whether the same factors are recruited in most cell types. Early identification of potential Sp1 binding sites highlighted their possible involvement in regulation [28]. In 1992, Cameron and colleagues used DNase I footprinting and gel retardation analysis to show that there are multiple factors that bind to distinct regions of the *INSR* promoter [27]. In particular, footprints were observed over the putative Sp1 sites. Lee et al., (1992) confirmed the binding of an Sp1-like protein to the *INSR* promoter by band-shift assays using HepG2 nuclear extracts [24]. In addition to the widely active Sp1 factor, tissue-specific transcription factors have also been implicated in regulation of the *INSR* promoter. In hepatocytes, the uncharacterized hepatocyte-specific transcription factor, HTFIR, and IR nuclear factors I and II (IRNF-I and IRNF-II) were found to be involved in *INSR* expression [24,25]. EMSA and UV crosslinking analysis identified the HTFIR in HepG2 and rat hepatocytes, while IRNFI and II were identified through DNase I footprinting and band shift assay. The high-mobility group protein A1 or HMGA1, which is upregulated in human cancers and in embryonic tissues, was also shown to bind to this promoter element and increase the expression of *INSR* using reporter assays and hormone binding studies in human IM-9 lymphocytes [29,30]. A minimal reporter containing *INSR* promoter- and first intron-derived C/EBP binding motifs, as well as the 1.8 kbp promoter construct, was shown to be activated in HepG2 cells by C/EBPα or C/EBPβ [31,32]. More recently, the TCF7L2 transcription factor, a mediator of wnt signaling, was found to bind to the *INSR* promoter and was shown to increase *INSR* expression three-fold when overexpressed in 3T3L1 cells [33].

Specific factors have likewise been identified that repress the proximal promoter. p53 overexpression partially represses this promoter in HepG2 and SAOS2 cells, dependent on GC-rich sequences located near -600; this regulation is suggested to involve antagonism of Sp1 and/or C/EBP. Indeed, the activation by C/EBPγ was partially blocked by co-expression of p53. The loss of wild-type p53 in many cancers was suggested to underlie the overexpression of IR noted in many cancer types [32]. While binding sites for the peroxisome proliferator-activated receptor-gamma (PPARγ) transcription factor have not been found on the promoter, PPARγ was shown to decrease *INSR* levels in HepG2 cells when overexpressed, potentially through disruption of Sp1, HMGA1, and/or C/EBPb binding to *INSR* [34]. In sum, these studies of a small promoter-proximal element of *INSR* suggested that this portion of the gene may play a role in integrating both basal and tissue-specific activities. Indeed, a promoter-proximal A-603G polymorphism is reported to have a protective effect in colorectal cancer [35]. This allele was investigated in a case of leprechaunism, where unknown mutations severely decreased expression of *INSR* [36].

**Figure 2:**
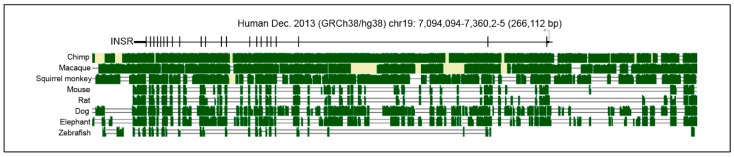
Sequence similarities of the *INSR* locus across vertebrates. Chimp, macaque, and squirrel-monkey show highest conservation to the human gene across the gene body and 5′ proximal promoter. Green indicates regions of conservation and yellow reflects an uncertainty in the similarity between DNA sequences. Single and double lines indicate unalignable bases due to a lineage-specific insertion/deletion or extensive divergence, respectively.

Evolutionary conservation can be a useful guide to identification of regulatory sequences, although they can change more rapidly than coding sequences and still retain function. Vertebrate Multiz alignment and conservation using the PhastCons program shows conservation among mammalian homologs in the 5′ UTR and across the 22 exons, including some conservation within intronic regions (Figure 2). Interestingly, regions 5′ of the TSS are less conserved than specific regions within introns 1 and 2. This pattern of conservation suggests the presence of internal *INSR* regulatory elements, which have not been fully analyzed in mammals. Studies of the Drosophila *INSR* homolog shows that internal enhancers are functional and combine to form an array of tissue- and signaling-specific control elements, pointing to a much more complex control of *INSR* than previously understood [22].

## 4. FOXO Feedback Regulation and Control of the Drosophila Insulin Receptor Gene

Dynamic cis-regulation of the insulin receptor gene is also observed in model organisms such as *C. elegans* and *D. melanogaster* [22,37]. The Drosophila Insulin-like Receptor protein (InR) was first described by Rosen and colleagues in 1985 [38], and after isolation of partial fragments, the entire *InR* cDNA and partial genomic DNA were cloned [39,40,41]. The amino acid sequence encoded by *InR* was found to be less than 40% identical to the human IR, but was suggested to be highly similar in function and structure. Similar to *INSR*, *InR* is a large gene that spans ~50 kb, of which 80% is comprised of introns. A distal promoter region adjacent to exon 1 is active in most developmental settings [42]; however, internal promoters that produce variant 5′ UTR sequences have also been identified [43]. Isoform-specific in-situ hybridization experiments imply that the *InR* isoforms may be differentially expressed in a spatial- and temporal-specific manner [44]. Differential promoter usage patterns have also been described in embryos, although follow-up studies in cell culture showed that the internal promoters have very low activity [22,43].

Similar to *INSR*, the *InR* gene is directly acted upon by a variety of factors that include the transcription factor dFOXO, ecdysone hormone, the dE2F1 transcription factor, and the retinoblastoma tumor suppressor protein (Rbf1) [5,7,22]. dFOXO is the Drosophila forkhead-related transcription factor, homologous to the mammalian FOXO proteins (FOXO1, 3, 4 and 6) and to the *C. elegans* DAF-16. dFOXO regulates *InR* expression through a feedback mechanism in response to nutrient availability: when nutrient levels are high, insulin-like peptide, or dILPs, activate the insulin signaling pathway by binding to InR [5,7]. This induces a signaling cascade in which the Akt pathway is activated and dFOXO is phosphorylated and is thereby retained in the cytoplasm. Phosphorylation prevents dFOXO from activating transcription of a variety of genes such as *d4EBP* and *InR* itself, representing a negative feedback loop. In contrast, when nutrient availability is low and the insulin signaling pathway is repressed, dFOXO is not phosphorylated, allowing for nuclear localization and activation of targets such as *InR*. In total, this feedback circuit ensures that conditions of low insulin signaling will result in production of insulin receptors, which are translocated to the cell membrane to act in response to future increases in nutrient levels [5,7]. Thus, the dFOXO feedback mechanism couples a direct regulation of dFOXO targets with an overall adjustment of signaling intensity through InR abundance.

Regulation of *InR* by dFOXO was first investigated by Puig and colleagues [5,7] in both cell culture and in vivo assays. They showed that an internal 1562 bp *InR* promoter (P2) fragment was activated upon dFOXO overexpression in S2 cells, and using chromatin immunoprecipitation, these authors showed that dFOXO directly binds this *InR* promoter. Several putative dFOXO binding elements were discovered within 200 bp of this *InR* P2 promoter. The dFOXO feedback effect is observed under conditions of nutrient limitation. Starvation of S2 cells for 6 h led to an increase in active dFOXO, with >20-fold increase in *InR* mRNA levels [5]. Higher protein levels were also observed. To test dFOXO regulation of InR in a physiologically relevant setting, adult flies were placed on a sugar-only diet for four days and were found to have a two-fold increase in *InR* mRNA levels. In contrast, dFOXO-deficient flies under similar conditions showed no change in the *InR* expression level, suggesting dFOXO is directly involved in the response to nutrients [5]. This regulation was also demonstrated in mammalian cells, where expression of mammalian FOXO1 induced an *INSR*-luciferase reporter. Expression of luciferase driven by the *INSR* promoter was observed to be dependent upon constitutive expression of FOXO1 in HEK293 cells, and expression was compromised when a FOXO response element was removed from the promoter. Band shift assays in vitro and ChIP-PCR experiments in vivo confirmed that this regulation was a result of direct binding of *INSR* by FOXO1. This FOXO1 regulation of *InR* was found to be conserved in mouse C2C12 muscle cells and Hepa 1–6 liver cells as well, indicating a conserved mechanism in mammals and flies [5]. Consistent with these findings, a number of cell types show reductions in *INSR* expression after in vitro treatment with insulin (summarized in Section 6).

This feedback circuit is consistent with the homeostatic regulation model suggested by Gavin and colleagues based on IR levels in cultured human lymphocytes, although at the time, the relative contributions of transcriptional and posttranscriptional regulation were unknown [45]. Evolutionary variations in insulin signaling have been found to underlie key differences at a population and species level; thus, one might ask whether FOXO control of the receptor is variable. Orengo et al. (2017) identified putative dFOXO binding sites 5′ of the internal promoter and noted that these predicted sites are evolutionarily variable at a species level, suggesting that this cis-regulatory variability may endow the gene with different feedback responsiveness [46].

The direct regulation of *InR* by dFOXO appears to represent only a part of the control paradigm, however. In cultured Drosophila cells, separate intronic enhancers mediate both positive and negative regulation of *InR* via dFOXO; some of these are through directly bound regions, but most appear to be mediated through indirect interactions involving downstream activators and repressors. Tissue-specific responses to dFOXO were also observed; some intronic enhancers are repressed by dFOXO in S2 cells but activated in Kc cells [22]. Overall, in these two cell types, the regulation of *InR* appears to be a complex integration of signals, involving at least nine distinct regulatory regions encompassing 13 kb of DNA. Both positive and negative signals are combined in a non-linear fashion, using coherent and incoherent feed-forward circuits [22]. Detailed studies of this sort involving the *INSR* have not yet been carried out in the mammalian system.

## 5. Tissue-Specific Expression of the Insulin Receptor

Tissue-specific differences in the expression of the insulin receptor protein in humans and rodents were first established by receptor binding assays and immunological approaches [47,48]. Differences in surface levels of the receptor can be affected by ligand-dependent receptor endocytosis. Binding of insulin enhances receptor internalization, the rate of which can differ among different cell lines; thus, measured differences in membrane-associated IR may reflect this dynamic process [49]. Total cellular differences, not just surface receptors, may reflect regulation at multiple levels, including transcriptional and post transcriptional effects. Examples of these levels of regulation have been observed in diverse settings. Longer mRNA half-life was correlated with higher levels of protein in HepG2 hepatocytes compared to IM9 lymphoblasts and normal human fibroblasts; here, transcriptional effects were not noted, as the rates of *INSR* transcription as measured by nuclear run-on assay were found to be the same [50]. Translational regulation via microRNAs is another level of control: miR424-5p has been reported to control *INSR* expression in HepG2 cells, and upregulation of this miRNA by palmitate is suggested to underlie dysfunctional insulin signaling in the liver [51]. Translational regulation via an internal ribosome entry site (IRES) in the *INSR* 5′ UTR may also influence protein expression in a cell-type specific manner [52]. In sum, the IR protein has been shown to be regulated at multiple post-transcriptional levels, a factor to be taken into account when considering the potential impacts of transcriptional differences.

At the level of mRNA, which may reflect transcription or turnover, early studies identified tissue-specific differences in levels in both human and rat tissues. Northern blot analysis of rat tissues showed liver to contain three-fold higher levels of *INSR* mRNA than muscle and brain [53]. A similar differential expression in liver was observed for human tissues [54]. These mRNA levels correlated tightly with measured IR protein levels in human liver, fat, and muscle samples [55]. Later studies in mice using whole body autoradiography showed a partial correspondence of these mRNA levels with protein, with high specific binding of insulin in liver, kidney, and brain, and lower binding in fat and muscle [48].

The tissue-specific differences found in these preliminary studies have been supported by more comprehensive analysis of *INSR* mRNA abundance in humans. The Genotype-Tissue Expression (GTEx) project characterizes the human transcriptome in 53 different tissues of adults and has created a reference resource of expression patterns in normal tissue [56]. *INSR* mRNA levels across multiple human tissue samples show a large range in abundance, from 1.58 transcripts per million (TPM) mapped reads in whole blood to 95.23 TPM in spleen. Most tissues, however, have a median expression level that is within one standard deviation of the global median of 21.3 TPM, consistent with the housekeeping function of *INSR* in maintaining cellular homeostasis. Spleen, ovary, and uterus, which have the highest *INSR* expression and whole blood, with the lowest *INSR* expression, lie outside this range, suggestive of additional tissue-specific roles that may involve increased insulin signaling sensitivity. Some aspects of differential expression appear to be conserved between vertebrates and invertebrates; in *D. melanogaster,* the *InR* level is highest in ovaries, with lower levels in the brain, similar to the human pattern [44,57]. It remains to be determined whether the tissue-specific differences in *INSR* mRNA rely on a single multifunctional enhancer [58] or multiple cis regulatory elements.

Importantly, *INSR* mRNA levels and protein expression are not always correlated. As assessed semi-quantitatively by immunohistochemistry, IR protein levels have been grouped into “undetected/low/medium/high” levels of expression, which correlated with RNA-seq data [56]. For example, while the high *INSR* mRNA and protein levels correlate in the ovary, the spleen shows high *INSR* mRNA expression, but low protein expression, whereas the reverse is true for the pancreas. In general, however, protein expression follows the measured mRNA. Differences observed in some tissues may reflect the posttranscriptional regulation discussed above.

## 6. Insulin Receptor Expression in Obesity, Insulin Resistance, and Diabetes Mellitus

Type 2 diabetes mellitus is a metabolic disorder in which resistance to insulin leads to altered glucose homeostasis and severe complications. The insulin receptor is a central node in this signaling cascade, and given the transcriptional feedback control from the FOXO transcription factor, changes in signaling effectiveness through insulin resistance may impact the transcription of *INSR*. Initial studies focused on the protein, as the gene sequences were unknown. From studies of insulin binding to IR on adipocytes and monocytes, hyperinsulemic, obese patients were observed to have lower concentrations of receptors. From a number of studies, an inverse relation was noted between insulin levels and cell surface receptors [59,60]. These levels responded to the nutritional state; a two-week fast increased monocyte IR receptor numbers, even as insulin levels dropped [61]. In light of this prior work, following the identification of the gene, it was worthwhile to examine *INSR* expression as a function of insulin signaling and glucose levels.

Several studies have tested the response of *INSR* mRNA levels to insulin or glucose in cultured cells. A marked reduction (by 60%) in *INSR* mRNA levels was seen in HepG2 liver carcinoma cells grown under lower glucose conditions (5.5 v. 25 mM) [62]. Similar lower steady-state levels of *INSR* were observed in IM9 lymphocytes grown under low glucose conditions [63]. Insulin treatment decreased *INSR* mRNA levels in a number of cell types assayed in vitro, including AR42J pancreatic acinar cells (to ~50% of previous levels), which was found to be concomitant with accelerated IR protein turnover [64]. In addition, modest reductions in *INSR* levels were noted in melanocytes in response to insulin treatment, with a reduction to one-third of the original level in primary human endometrial decidual cells [23,65]. *INSR* expression in endometrial cells is highly dependent upon FOXO1 activity, and the insulin-dependent decrease was dependent on PI3K signaling, consistent with the regulation of FOXO1 by phosphorylation [65]. These studies are representative of a wide-range of investigations in which acute exposure to insulin induces a decrease in steady-state *INSR* mRNA levels, consistent with the negative feedback loop mechanism. A reduction in steady state levels of the receptor may help to maintain dynamic sensitivity at higher levels of the ligand. However, such feedback may not be a universal response. IM9 lymphocytes did not show changes in *INSR* levels upon nanomolar to micromolar insulin treatment [66]. In addition, primary human umbilical vein-derived endothelial cells (HUVEC) did not show changes in *INSR* mRNA levels when treated with 1 nmol insulin, a level that induces physiological changes in these cells. However, HUVEC cells from patients with gestational diabetes showed modest reductions in transcript levels, affecting specifically the A isoform [67]. Other studies similarly point to differences in the sensitivity of *INSR* mRNA levels to exogenous insulin levels; thus, a FOXO1 negative feedback mechanism may be less important in some cells [68,69,70,71].

Despite the acute effects found for cultured cells upon treatment with exogenous insulin, changes in steady state levels of *INSR* mRNA do not show a uniform pattern in different tissues from patients either with established Type 1 or Type 2 diabetes or with predisposing conditions including obesity and insulin resistance. For instance, early studies identified no effect of acute insulin treatment on *INSR* in skeletal muscle in both insulin resistant, nondiabetic subjects as well as in an insulin-sensitive group. Human cell types analyzed in these studies included skeletal muscle, adipose tissue, peripheral mononuclear leukocytes, and pancreatic islet cells [72,73,74]. Steady state levels of *INSR* in muscle were, however, significantly lower (approximately one-half) in the insulin-resistant group [75]. In a different human study, although no direct correlation between *INSR* levels and glucose tolerance was observed in liver, muscle, and adipose tissue, there was a strong negative correlation between adipose *INSR* levels and BMI, as well as a positive correlation between adipose *INSR* levels and measured insulin tolerance [54]. Consistent with the overall metabolic condition influencing steady-state levels, *INSR* mRNA levels measured in whole blood samples from children were modestly higher in overweight vs. normal weight boys, but no difference was found between these two groups of girls [76].

In model systems, a variety of approaches have been taken to assess the possible impact on levels of *INSR* mRNA (as well as other genes) by disease state models. The levels of *INSR* mRNA measured in liver and skeletal muscle of pancreatomized, insulin resistant diabetic Sprague Dawley rats were no different from those of control rats [77]. Skeletal muscle insulin receptor levels of zebrafish remained unaltered under hyperglycemic conditions, and diet-induced hyperglycemic Drosophila showed no detectable changes in the insulin receptor expression when compared to control flies [78,79]. To determine more transient effects, in a euglycemic model of the mouse, insulin administration led to perturbed expression of genes in the insulin signaling pathway in skeletal muscle and liver within three hours, while *INSR* levels themselves were unchanged. Interestingly, IR protein levels were depressed by this treatment in muscle, but not in liver, highlighting additional tissue-specific levels of IR protein regulation [9].

**Figure 3:**
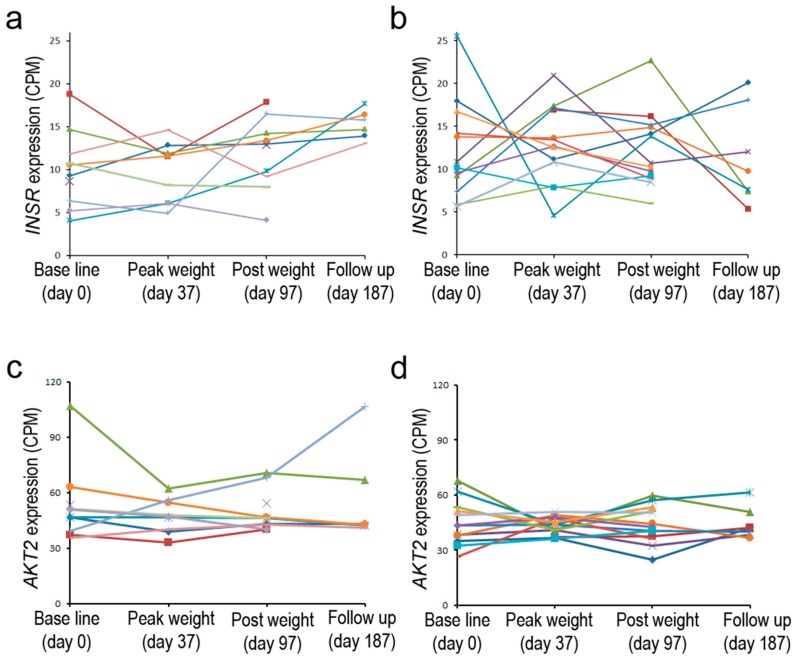
*INSR* expression in non-insulin-resistant and insulin-resistant subjects. Steady-state levels of *INSR* or *AKT2* mRNA observed in peripheral blood monocytes (PBMC) from (**a**,**c**) overweight to obese, non-insulin resistant and from (**b**,**d**) overweight to obese, insulin-resistant individuals during a course of weight gain and subsequent loss. Mean levels in baseline *INSR* levels (**a**,**b**) were similar in these two classes; however, there were more than >2X changes in the levels in the insulin-resistant category after a period of weight gain. *AKT2* levels showed less variation in baseline levels and during weight changes for both of these groups (**c**,**d**) (data from [80]).

These prior studies examined the expression of *INSR* at a single time point or shortly after administration of insulin; in contrast, a recent personalized medicine study tested the impact of moderate weight gain and loss in overweight individuals who differed in insulin sensitivity [80]. Gene expression at various time points was monitored by RNA-seq in peripheral blood monocytes (PBMC). Baseline *INSR* expression ranged from 5 to 25 CPM in different individuals and was similar for both groups. After a period of weight gain, up to two-fold increases or decreases were noted, particularly for individuals belonging to the insulin-resistant group. More modest changes were seen in the insulin-sensitive group (Figure 3). The greater observed dynamics of gene expression in one group may reflect the impact of insulin resistance on transcriptional control of this gene. The inherent differences in expression of the gene in the baseline state may furthermore indicate that genetic and/or environmental factors can have a significant influence on the expression of *INSR*; such subject-to subject variability in liver, adipose, and muscle tissue was also noted in early studies in which the highly quantitative S1 endonuclease assay was employed [54]. It is interesting that *AKT2*, a gene that is downstream in the insulin signaling pathway, showed a less dynamic pattern of expression in the two subject groups [80]. Because of the small number of subjects and lack of technical replicates, the statistical significance of these patterns was uncertain. It still remains to be determined whether changes in receptor expression are associated with the pathophysiology of the disease, particularly because the *INSR* expression is measured in PBMC, not in central metabolic target tissues. However, the observed differences in baseline expression are not likely to be solely due to genetic factors; steady-state *INSR* mRNA levels can be dynamically regulated in a variety of cell types by exogenous factors as discussed below. Overall, there are examples in which differences in metabolic status may have a long-term effect on steady-state *INSR* expression, but the reproducible acute effects noted for cultured cells are generally not reliably found in most tissues sampled from study subjects. Complex adaptive mechanisms may underlie the lack of a simple correlation in many cases.

## 7. Evidence for Specific Regulation of *INSR* Transcription by Endogenous and Exogenous Stimuli

Glucocorticoids have been linked to glucose homeostasis, and extended treatment with synthetic glucocorticoids such as prednisone can lead to insulin resistance. Therefore, these hormones have been tested for their impacts on *INSR* expression. Dexamethasone treatment induced up to a four-fold increase in *INSR* mRNA levels in cultured IM9 lymphocytes. Nuclear run-on experiments showed that this was a direct consequence of an increased transcriptional rate, as half-life and nuclear export were not affected [63,81]. A similar dose-dependent response in *INSR* levels was noted in response to hydrocortisone treatment in these cells [66]. Interestingly, these changes in transcript levels in IM9 cells were dependent on glucose levels in the culture medium, which was typically held at 25 mM glucose; cells grown under low (5.5 mM) glucose did not show an increase, suggesting the convergence of multiple interdependent signaling pathways on the *INSR* gene [63]. Dexamethasone has been found to induce *INSR* expression in other cell types as well, such as UMR 106-01, an osteogenic sarcoma cell line [82]. This hormone also impacts relative levels of *INSR* splice variants in HepG2 hepatocytes [83]. While dexamethasone has similar effects in these cell lines, not all tissues responded identically in an intact animal. Dexamethasone treatment of rats induced three-fold *INSR* expression in the liver, but no change was observed in adipocytes, indicating tissue specificity [84].

Other steroid hormones can induce the expression of *INSR*. Androgen treatment of cultured HEp-2 epidermoid carcinoma cells induced expression of the gene, by approximately three-fold [85]. Treatment of MCF-7 human breast cancer cells with estradiol induced transcription of *INSR* within ten minutes as measured by Gro-seq, suggesting direct effects [86]. In contrast, HepG2 or IM9 cells showed no change in steady-state levels of *INSR* upon treatment with estradiol, suggesting that this hormone may function in a cell-type specific manner [87,88]. 17*β* estradiol treatment of U-937 cells reduced the expression from a reporter gene carrying a 1.6 kb *INSR* promoter fragment [89]. 1,25-dihydroxy vitamin D3, another steroid hormone, induced an *INSR* reporter (1.6-fold) in U-937 human promonocytic cells. In this case, a minimal region 5’ of the insulin receptor gene (−271 to −876) was shown to contain vitamin D response elements and was regulated by vitamin D3 treatment, making this hormone the only one with identified functional cis-regulatory sites on *INSR* [90,91].

Progesterone treatment of primary human endometrial stromal cells induced *INSR* expression by ten-fold, half of which was due to FOXO1-dependent signaling [92]. An earlier study noted that synthetic progestin treatment of the T-47D human breast cancer cell line induced *INSR* expression by two-fold [93]. Collectively, these studies indicate that the *INSR* gene is regulated by distinct hormones in a tissue- and culture condition-dependent manner (summarized in Table 1). If the action of all of these hormones is direct, there are likely to be separate cis-acting regulatory elements with corresponding motifs for the respective nuclear hormone receptors. In these studies, the impact of these hormones was generally stimulatory; however, more detailed molecular analysis of individual cis regulatory elements of the Drosophila *InR* gene indicates that there may also be both positive- and negative-acting enhancers contained within the *INSR* locus [22].

The predicted complex transcriptional regulatory circuitry of the human *INSR* gene likely integrates inputs from additional signaling pathways. For instance, berberine, an alkaloid used in traditional Chinese medicine to treat diabetes, upregulates *INSR* expression in a protein kinase D-dependent manner in cultured liver cells [94]. Other compounds have an inhibitory effect; forskolin, a regulator of intracellular cAMP levels and protein kinase A, causes a decrease in *INSR* expression in IM9 cells. This change, as noted for dexamethasone treatment, was sensitive to the amount of glucose under culture conditions [63]. In addition to hormones and xenobiotics, viruses can also alter the abundance of *INSR* transcripts. Epstein Barr virus-transformed Burkitt lymphoma cell lines had higher levels of surface-bound IR protein and *INSR* mRNA compared to untransformed lymphoma cells [95]. Infection of endothelial cells with Kaposi’s sarcoma-associated herpes virus (HHV-8) was associated with five- to ten-fold increases in the expression of *INSR* [96].

**Figure 4:**
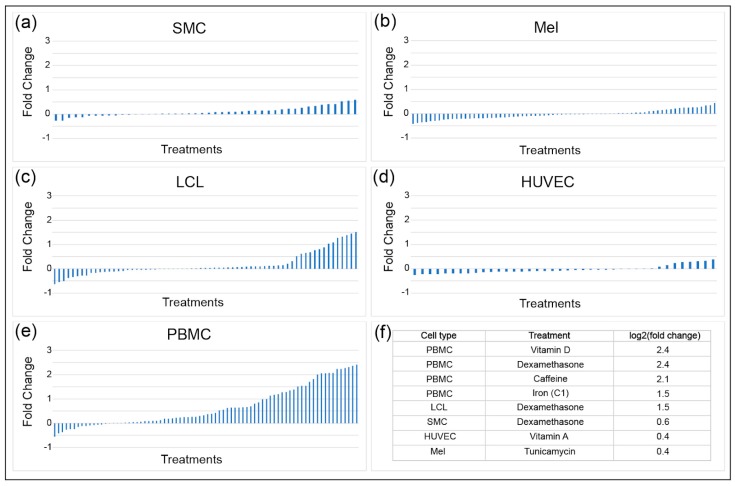
Dynamics of *INSR* expression in response to environmental perturbations. *INSR* mRNA levels were assessed in five different human cell types: (**a**) smooth muscle cells (SMC); (**b**) melanocytes (Mel; (**c**) lymphoblastoid cell lines (LCL); (**d**) human umbilical vein endothelial cells (HUVEC); and (**e**) peripheral blood mononuclear cells (PMBC). Blue bars represent *INSR* log_2_ (fold change) expression observed in cultured cells after a 6-hour treatment. Overall dynamic ranges in *INSR* expression differed among different cell types; (**f**) Examples of treatments leading to the highest levels of induction are listed in the table, including the top four treatments for PMBCs and the top treatment for LCL, SMC, HUVEC, and melanocytes (data from [23]).

Environmental perturbations such as hormones, metals, dietary components, drugs, and other environmental contaminants have been shown to impact *INSR* mRNA levels. To determine the magnitude and direction of transcriptional effects of these molecules on gene expression, a gene by environment (GxE) study used 50 treatments on five cell types obtained from fifteen individuals [23]. A high-throughput two-step RNA-seq approach was used to determine transcriptional responses to treatments. The five cell types tested were human umbilical vein endothelial cells (HUVEC), lymphoblastoid cell lines (LCL), melanocytes (Mel), smooth muscle cells (SMC), and peripheral blood mononuclear cells (PMBC), with three samples from each tissue from each individual. *INSR* expression in SMC, melanocytes, and HUVEC was least responsive to the various treatments, with changes in expression ranging from a log_2_ fold change of −0.5 to +0.5 (Figure 4). LCL and PBMC had a much more dynamic change in expression, reaching up to a 5.3-fold increase in *INSR* in response to treatments such as vitamin D and dexamethasone for PBMC. Cell-type specific responses were observed for some treatments, such as for caffeine: PBMC had the greatest increase in *INSR* expression in response to caffeine (up to four-fold), while SMC had very modest increases (up to only 1.3-fold). Treatments that resulted in decreased *INSR* expression included selenium in LCL, insulin in melanocytes, and specific vitamins in PBMC. The treatments that caused the greatest decreases differed by cell type, indicating that distinct regulatory pathways impact *INSR* expression in different cell types. However, some treatments such as dexamethasone induced expression independent of cell type, pointing to some universally active regulatory circuitry for *INSR*.

These studies demonstrate that levels of *INSR* mRNA are sensitive to intrinsic or extrinsic perturbations, which manifest in a cell- and context-dependent manner. Signals regulating these responses may feed through FOXO-dependent or -independent pathways. Other transcription factors that have been functionally characterized as interacting with *INSR* cis-regulatory regions, such as Sp1, are not known to be dynamically regulated; therefore, there may be additional regulatory factors still to identify. If *INSR* regulation resembles that found on developmentally regulated, tissue-specific genes, it is possible that the enhancers of this gene will respond to multiple interconnected signaling cascades.

This overview of *INSR* expression in cells and intact organisms indicates that there are regulatory elements in *INSR* that drive tissue- and developmental stage-specific regulations, which are impacted under pathological conditions. In addition to diabetes-related conditions, expression of the insulin receptor is transcriptionally upregulated in many types of cancer, including breast, prostate, bladder, and thyroid. The risk of liver cancer is particularly elevated in diabetics, which may reflect the mitogenic effects of insulin, especially on cancer cells expressing predominantly the IR-A isoform [97,98]. Whether overexpression represents abnormal activation of conventional regulatory circuits or loss of repression (for example, through loss of p53) is not generally understood [32]. In sum, extensive previous work has delimited conditions under which *INSR* is differentially expressed, but we have very limited knowledge about the DNA elements that dictate the responses. To gain a better insight into the likely relevant control regions, we summarize below our understanding of the insulin receptor chromatin landscape of *INSR* and sequences associated with population-level variation and disease.

## 8. Omics Approaches to Studying the *INSR* Chromatin Landscape

**Figure 5:**
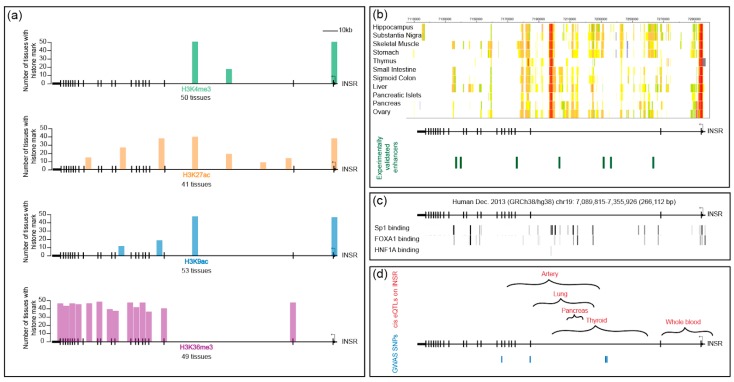
Histone marks, representative transcription factor binding sites, chromatin states, and location of informative SNPs on the *INSR* locus (**a**) Location of histone marks on the *INSR* locus. The X-axis represents the *INSR* gene with the promoter on the right, indicated by a black arrow. The Y-axis represents the number of tissues with the particular histone mark. Activation marks including H3K4me3, H3K27ac, and H3K9ac overlap at the promoter and a site with promoter-like features in the center of the gene, in intron 2. These marks are also found in a tissue-specific pattern elsewhere on the gene. H3K36me3 marks are concentrated on the 3’ end. Data obtained from the Roadmap Epigenomics Project [108]; (**b**) Chromatin states shown for the *INSR* locus in eleven tissues. Red: active TSS, orange: active enhancer, yellow: weak enhancer, lime green: transcribed and weak enhancer, gray: repressed polycomb, white: quiescent/Low. Promoter-like regions are indicated in red and overlap with the annotated *INSR* promoter, as well as with a region of intron 2 that does not appear to generate significant transcriptional starts. Tissues were selected to illustrate examples of tissue-specific and universal marks. The skeletal muscle tissues are from females, and stomach tissue is specifically from smooth muscle [108] (25 state model shown). Below, green lines indicate locations of experimentally validated enhancers from whole-genome STARR-seq data in the LNCap prostate tumor cell line [108]; (**c**) Binding sites of Sp1, FOXA1, and HNF1A. Vertical lines indicate bound sites from ChIP-seq data. The gradient from gray to black indicates the number of tissues in which the binding event was observed (data obtained from [42,109]); (**d**) Significant cis eQTLs found on the *INSR* locus. Brackets show locations of tissue-specific clusters of eQTLs found in artery, lung, pancreas, thyroid, and whole blood. Locations of four GWAS SNPs associated with polycystic ovary syndrome, height, thyroid hormone levels, and triglycerides are indicated (data obtained from [56]).

Chromatin accessibility and modifications are often used as proxies for regulatory regions, and indeed they often closely correlate with neighboring gene activity. In diverse tissues, the *INSR* locus is enriched for active histone marks, including H3K27ac, H3K4me3, H3K9ac, and H3K36me3 (Figure 5a). The annotated *INSR* TSS is enriched in H3K4me3, a mark typically found at promoters. An additional H3K4me3 peak is found at the center of the gene, within the large second intron. Both of these peaks are found in all tissues, and they correspond with a ChIP peak for RNA Pol II (not shown here). Cap analysis of gene expression (CAGE) data indicate that a complementary transcript arises from the internal TSS, but the transcript is apparently unstable because it is not detected in RNA-seq datasets; its significance is therefore currently unclear. RNA Pol II ChIA-PET (chromatin interaction analysis by paired-end tag sequencing) data obtained using MCF-7 and HCT116 cells show an interaction between the *INSR* promoter and the internal promoter-like sequence within intron 2. Long-range interchromosomal interactions play a functional role in modulating gene expression; the functional significance of the interactions observed at the *INSR* locus remains to be tested [99,100] (references therein).

Two other activation marks, H3K27ac and H3K9ac, co-occur with these promoter-like regions in most tissues and also show tissue-specific peaks within the body of the gene. They overlap in some but not all positions. Indeed, a survey of cellular enhancer-like sequences confirm that enhancers vary in patterns of chromatin modification and may have different levels of specific marks [101] (references therein). Consistent with other studies, H3K36me3, which is linked to transcriptional elongation and is associated with the 3′ regions of genes, is enriched on the latter half of *INSR* [102]. Differential H3K36me3 modification is associated with exon definition, and this region of the gene includes exon 11, which is alternatively spliced. In contrast to the rich presence of activation-associated marks in most tissues, few cell types feature the repressive H3K27me3 mark on the body of the gene, suggesting that *INSR* is not typically regulated by the PRC2 polycomb repressive complex (not shown). H3K9me3, a mark associated with heterochromatin, appears in diverse patterns on the *INSR* gene across different cell types and tissues (not shown). In many embryonic stem cell lines, overall H3K9me3 levels are quite low, whereas many tissues exhibit localized peaks of this mark toward the 3′ end of the gene. A few cells, such as mammary epithelial cells, show widespread marks across the body of the gene. In general, however, observed H3K9me3 peaks are low and scattered. H3K9me3 domains have been shown to be important for impeding reprogramming and maintaining cellular identity [103], so the depleted levels in ES cells may reflect differential regulation of this gene.

Open chromatin and eukaryotic enhancers are often distinguished by DNase I hypersensitive sites (HS). In surveys of a wide spectrum of immortalized human cell lines as well as tissues, clusters of DNase I HS are distributed fairly evenly across the *INSR* locus [42] (not shown). With the exception of T cells, hypersensitive regions overlap the annotated promoter and the internal promoter-like chromatin region at the 3′ end of the second intron. Cell type-specific HS regions correlate with active histone modifications, consistent with previous observations [104]. Interestingly, a large stretch (>50 kbp) of the genome 5′ of the promoter lacks significant DNase I sites, another piece of evidence suggesting that human *INSR* regulatory regions lie within the transcription unit, similar to the arrangement in Drosophila [22].

The chromHMM-based chromatin state assignment for *INSR* integrates diverse types of chromatin data to predict putative regulatory regions. Using this predictive tool, candidate enhancers are predicted within the first three introns of *INSR*, and their spatial distributions vary in a tissue-specific manner (Figure 5b). Some of these predicted regulatory regions overlap with the binding of the Sp1 activator, the pioneer factor FOXA1, and the tissue-specific hepatocyte nuclear factor 4 (HNF4/1A) (Figure 5c). Sp1 binding sites can be found throughout the body of the gene, which is not consistent with a general notion that this factor is largely active at promoters. If these sites are functional, they may play a role in the function of internal putative enhancers. Binding sites across the *INSR* locus are also found for FOXA1, which has been characterized as a pioneer factor, binding to closed chromatin regions and facilitating access of other regulatory factors. Its binding to *INSR* may open up and maintain enhancers, potentially playing a role in tissue-specific elements. For instance, the tissue-specific transcription factor HNF4/1A is found to bind to one site within intron 2 in some tissues and may contribute to some of the tissue-specific effects discussed above. Interestingly, FOXA1 and HNF4/1A can synergize to facilitate reprogramming of hepatocytes [105]. Consistent with the paucity of activating marks outside of the gene body, chromHMM-predicted enhancers are absent in 5′ and 3′ regions of the gene.

Few studies have attempted to validate the functional activity of the predicted *INSR* enhancers. One exception is a study that employed cell-type specific, whole-genome STARR-sequencing. This method assesses the transcriptional potential of small fragments of the genome that are inserted into reporter genes. Assayed in a prostate cancer cell line, >90,000 regions from across the genome exhibited regulatory activity, mostly from intronic or intergenic regions [106]. Analysis of this data set revealed that four regions within the second intron and three regions in other introns of the *INSR* gene contained significant enhancer activity in this cell line (Figure 5b). Some of these regulatory regions overlapped with H3K27ac and H3K4me1 active histone marks.

Analysis of population-level variation provides additional lines of support for *INSR* regulatory regions within the gene body, overlapping with areas identified from chromatin studies. GTEx datasets indicate that multiple eQTLs (expression quantitative trait loci) are present within the first two-thirds of the gene body; data from different tissues indicate that there may be some differentiation of tissue-specific elements (Figure 5d). The relevant SNPs may be linked to or may themselves be binding sites for regulatory factors within *INSR* enhancers. Consistent with the possibility that cis-regulatory changes may result in overt phenotypes, four loci within the gene body were associated in genome-wide association (GWA) tests to polycystic ovary syndrome, height, thyroid hormone levels, and triglycerides [56]. These have not been assessed for impact on the expression of *INSR* mRNA or protein, however. Interestingly, additional assessment of GWA from BioBank, which assesses genetic and clinical data from 500,000 individuals in Great Britain, shows association of SNPs centered in the second intron and occasionally in the 3′ UTR, with related phenotypes including height, weight, BMR, hip circumference, arm, and whole body impedance, which are also consistent with the metabolic and growth pathways regulated by IR (www.nealelab.is/uk-biobank). In sum, population sequence variation present within the human *INSR* gene suggests that there are important regulatory regions within the body of the gene overlooked in the initial focus on the 5′ proximal sequences. In some cases, these variant sequences appear to correlate with differential expression of the gene, which may be a function of transcriptional control, although changes that affect splicing and inherent transcript stability cannot be ruled out. In addition, numerous highly suggestive physiological phenotypes are linked to variation within this gene, which may again reflect altered transcriptional properties of *INSR*, although these remain to be validated experimentally. Besides genetic variation observed in the putative regulatory sequence of *INSR*, a study of Dutch Hunger Winter cohorts prenatally exposed to malnutrition suggest that DNA elements in the *INSR* regulatory region are epigenetically programmed during early development to affect metabolic processes in later life [107].

## 9. Evolutionary Adaptation of Insulin Receptor Signaling

The complex transcriptional regulatory regions of the Drosophila *InR* gene and the presumably similarly complex controls for the mammalian *INSR* gene provide an extensive landscape that may underlie evolutionary transitions. Indeed, work from model systems has provided evidence for evolutionary alterations to the insulin receptor and its regulation. With clear functional consequences, specific mutations in coding sequences of the insulin receptor gene generate important physiological differences. For instance, at the population level, protein changing alleles of *InR* appear to dictate latitude-dependent differences in Drosophila body size [110]. The extraordinary starvation resistance of cave populations of *Astyanax mexicanus,* the Mexican tetra, has similarly been linked to mutations affecting the insulin binding of the receptor [111]. Estimations of evolutionary selection of the *InR/INSR* gene show strong purifying selection on the part of the gene encoding the hormone-binding portion of the protein, with evidence of more recent divergence in the cytoplasmic kinase domain [112]. Remarkably, the overall level of selection of genes comprising the insulin signaling pathway shows a similar bias in invertebrates and vertebrates, with genes acting downstream of *InR/INSR* being the most constrained, suggesting that there may be more genetic leeway for novel variants in *InR/INSR* [113,114].

In other systems, evolutionary adaptations associated with insulin signaling play key roles in evolved features, although the direct role of *InR* transcription is yet to be established. For instance, the control of body size during development of Drosophila is dependent on nutritional signaling, and those tissues that are resistant to such allometric scaling have been shown to exhibit reduced sensitivity to insulin signals [115]. In contrast, the hypersensitivity of the larval tissues that become the “weapon” of the male rhinoceros beetle has been traced to an increase in insulin signaling sensitivity [116]. Insulin signaling has also been demonstrated to be critical for developmental plasticity with respect to ovariole number in Drosophila. Variable insulin signaling is suggested to underlie the evolutionary differences in body size and ovariole number in *D. melanogaster* vs. *D. sechellia* [117]. All of these studies indicate that the expression or activity of the insulin receptor provides the type of variation that is selected for fixation of unique traits at the population and species levels.

Gene duplication of the *InR* gene as observed in planthoppers represents another pathway in which insulin signaling is evolutionarily adapted. In this system, FOXO-dependent control of wing development is differentially regulated by two paralogs of the receptor to drive either the short-wing (localized) or long-wing (dispersive) body phenotype of this insect. Nutritional signals from the host plant can dictate the resulting wing polyphenism [118,119,120]. Another type of phenotypic plasticity is that of environmental sex determination. Many species of reptiles, including painted turtles, feature temperature-dependent developmental switches that favor development of one sex at lower temperatures (termed temperature-dependent sex determination, or TSD). Comparisons of transcriptomes from such species, compared to sister species using genotypic sex determination (GSD), show that certain genes, including *INSR*, exhibit temperature-sensitive transcription levels during embryogenesis only in TSD species, i.e., not in GSD species. *INSR* plays a role in the expression of the sex-determining gene *Sry*; thus, the evolutionary changes in the elements responsible for embryonic regulation of *INSR* may contribute to this developmental switch [121]. Overall, given the similarity of insulin signaling components across metazoans, there is ample reason to believe that the population variants affecting enhancers in the human gene are functionally relevant and underlie important physiological differences as well.

## 10. Conclusions

Despite the measurable dynamic properties of the *INSR* transcript, the physiological significance of transcriptional regulation of the insulin receptor gene has been unclear for many years. From the earliest molecular characterization of the locus encoding the IR, *INSR* was originally described as a housekeeping gene, consistent with properties of the basal promoter and the gene’s widespread expression [18]. Despite the proven dynamics of IR protein expression in response to signaling, the small fraction of receptors bound under physiological concentrations of insulin [122] may have supported a view that the actual levels of the receptor may not be particularly important. In addition, the known significant role of dynamic insulin levels in stimulating the pathway has led to a focus on downstream effects, rather than transcription of the *INSR* gene. Certainly, this view has begun to change with new molecular discoveries; the identification of a direct feedback loop from FOXO transcription factors provides a mechanism for adaptive control of the receptor, something foreshadowed by the pioneering work of Roth and colleagues [45]. The flood of genome-wide data, both in the form of transcriptomics, revealing dynamic modulation of the gene in certain settings, as well as genome-wide association signals mapping to SNPs within likely *INSR* enhancers, have changed this picture. The detailed experimental identification of a complex signaling system in the Drosophila *InR* gene has provided a first comprehensive view of regulation of a metazoan insulin receptor gene, likely mirroring the complexity of the mammalian gene. Work with model systems can provide a pathway for connecting population-level data, as well as specific developmental and physiological phenotypes of enhancer variants [22]. The challenge for the future will be to understand, at a quantitative level, the physiological significance of cis-regulatory variation in *INSR*, an undertaking that will necessitate integrated experimental and computational approaches. Such interdisciplinary work will yield important fruits for the fields of precision medicine, disease research, and developmental and evolutionary biology.

## Figures and Tables

**Table 1:** Summary of transcriptional response of *INSR* to steroid hormone treatments in human cell lines.

Treatment	Cell Line	*INSR* mRNA Changes	Ref.
Dexamethasone	IM9, UMR 106-01	increase	[63,81,82]
Hydrocortisone	IM9	increase	[66]
Androgen	HEp-2	increase	[85]
Estradiol	MCF-7	increase	[86]
Estradiol	HepG2, IM9	no change	[87,88]
Estradiol	U-937	decrease (reporter gene)	[89]
Vitamin D3	U-937	increase (reporter gene)	[90,91]
Progesterone	Endometrial stromal cells, T-47D	increase	[92,93]
